# A pediatric case report and literature review of facioscapulohumeral muscular dystrophy type1

**DOI:** 10.1097/MD.0000000000027907

**Published:** 2021-11-24

**Authors:** Ting Xiao, Haiyan Yang, Siyi Gan, Liwen Wu

**Affiliations:** a Department of Pediatric Neurology, Xiangya Hospital, Central South University, Changsha, Hunan, PR China; b Department of Pediatric Neurology, Hunan Children's Hospital, Changsha, Hunan, PR China.

**Keywords:** early-onset, extramuscular multisystem diseases, facioscapulohumeral muscular dystrophy, infantile

## Abstract

**Rationale:**

Early-onset facioscapulohumeral muscular dystrophy (FSHD) is defined as facial weakness before the age of 5 and shoulder weakness before the age of 10. Early-onset facioscapulohumeral muscular dystrophy is relatively rare in the clinic. This onset is relatively early, the symptoms are serious, and it is likely to be accompanied by retinal vascular disease, sensorineural deafness, epilepsy and other extramuscular multisystem diseases. We report the clinical characteristics of 2 patients with early-onset facial and shoulder brachial muscular dystrophy to improve clinicians’ understanding of this particular condition.

**Patient concerns:**

We report 2 pediatric patients with FSHD type 1. Patient 1 is an 11-year-old boy with reduced facial expression for 9 years and proximal muscle weakness for 6 years. Patient 2 is a 4-year and 6-month-old girl with developmental delay for 3 years and facial weakness for 1 year.

**Diagnosis:**

According to the clinical manifestations and molecular genetic testing (such as Southern blot analysis), the patients were diagnosed with early-onset FSHD1.

**Interventions:**

The patients received cocktail therapy (vitamin B1 tablets, vitamin B2 tablets, vitamin B6 tablets, vitamin C tablets, vitamin E tablets, idebenone tablets, etc.) to improve their muscle metabolism.

**Outcomes:**

Both patients’ condition did not improve after being given cocktail treatment. According to a recent follow-up, the symptoms of facial weakness and proximal muscle weakness were aggravated.

**Lessons:**

Early-onset FSHD presents early and has frequent systemic features, and it is a severe subtype of FSHD. Early identification and genetic diagnosis should be performed to improve patient prognosis.

## 1 Introduction

Facioscapulohumeral muscular dystrophy (FSHD, OMIM: 158900) is the third most prevalent muscular dystrophy. Age at onset of FSHD varies from infancy to late adulthood. However, most patients present in the second or third decade of life, the classical FSHD phenotype. Early involvement of facial and scapular muscles, subsequent involvement of proximal muscles (including the biceps and triceps), and final involvement of the pelvic girdle muscles are typical manifestations of FSHD. Early involvement of the tibialis anterior muscle is an exception. The bulbar, extraocular, masseter, temporalis, and respiratory muscles are usually not involved.^[1–4]^ Infantile or early-onset FSHD has rarely been reported. Infantile or early-onset is estimated to occur in approximately 10% of all patients with FSHD. Early-onset FSHD is defined as facial weakness before the age of 5 years and shoulder weakness before the age of 10 years.^[2]^ The pattern of muscle involvement in early-onset FSHD is similar to the classical FSHD phenotype, but with severe muscle weakness and frequent systemic features.^[1–3]^

## 2 Case report

### 2.1 Patient 1

Patient 1 was an 11-year-old boy. At the age of 2, his facial expression was reduced, with stiff facial movements, unable to show his teeth and facial diplegia. At the age of 5, there was no obvious cause for his movement retrogression, which involved an abnormal gait, uncoordinated gait, swinging, easy wriggling and poor physical strength compared to his peers. His bilateral shoulder joints were asymmetrical, and his right shoulder was lower. He was observed to have scoliosis and scapular winging. The proximal muscle strength of his upper limbs was grade 4, his distal muscle strength was normal, and his muscle tension was slightly high. Muscular atrophy was mainly seen in the shoulder, back, and proximal extremities, his bilateral knee tendon reflexes were weakened, and his bilateral achilles tendon reflexes were not evident.

His creatine kinase was 666.7 U/L (normal range 50–250 U/L). His cranial MRI was normal, and the MRI of his thigh muscles showed bilateral lateral femoral, rectus femoris, and left semimembranosus, and the long head of the biceps femoris was significantly atrophied. His audiovisual evoked potential was normal. Electromyography showed that the motor unit potentials of the quadriceps femoris on both sides were low and that the time limit was narrow. There was a moderate denervation potential, which showed pathological interference when contracting vigorously: the right deltoid and biceps had normal motor unit potentials. The scope and time limit were narrow, a small amount of denervation potential was seen, but recruitment was complete when contracting vigorously: The bilateral gastrocnemius muscles were normal. The nerve conduction velocity was normal.

A muscle biopsy showed mid muscle fiber size, no obvious necrosis or regeneration of the muscle fibers, a little inflammatory cell infiltration in the interstitium, and mild hyperplasia of the connective tissue. His electromyography and muscle biopsy showed myopathy changes. Southern blot analysis of DNA from the patient's blood sample with p13E-11 revealed an abnormal 7.3 kb *EcoRI* DNA fragment, which led to a diagnosis of FSHD1. The patient receive cocktail therapy (vitamin B1 tablets, vitamin B2 tablets, vitamin B6 tablets, vitamin C tablets, vitamin E tablets, idebenone tablets, etc.) to improve his muscle metabolism. The patient's symptoms did not improve during follow-up 1 month after discharge. According to a recent follow-up, the patient's facial weakness and proximal muscle weakness were aggravated.

**Figure 1:**
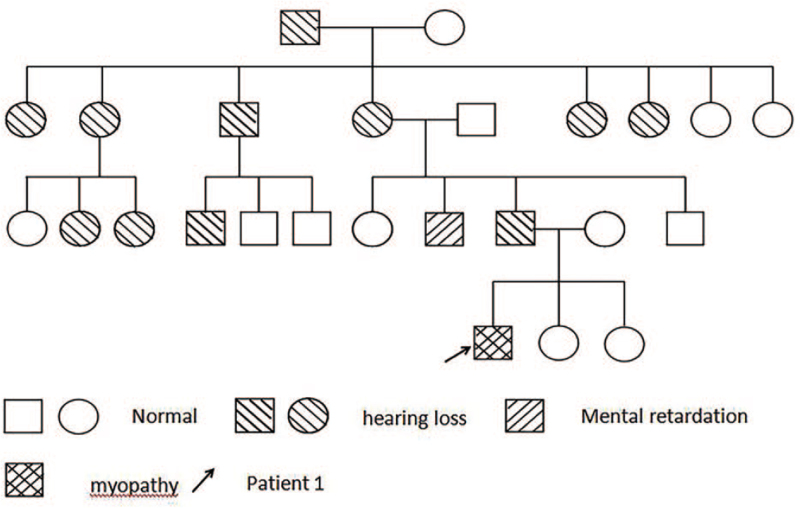
Family history of patient 1.

**Figure 2:**
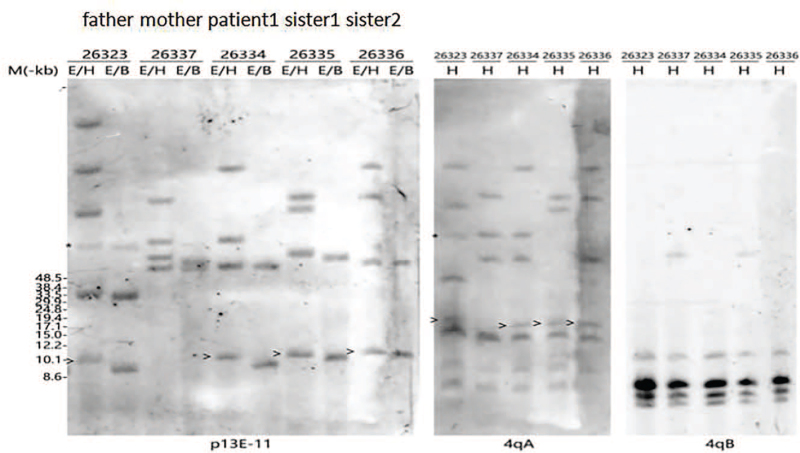
Southern blot analysis with p13E-11 in the family of patient 1. E: genomic DNA digestion with EcoRI restriction enzyme; H: genomic DNA digestion with HindIII restriction enzyme; E/B: genomic DNA digestion with EcoRI/BlnI restriction enzymes; E/H: genomic DNA digestion with EcoRI/HindIII restriction enzymes.

The family history (shown in Fig. 1) showed that there were 11 family members who had hearing loss and his uncle had mental retardation. Both of his sisters have facial weakness. Southern blot analysis with p13E-11 in this family revealed an abnormal 7.3 kb fragment in this boy, his father and his affected sisters (shown in Fig. 2).

### 2.2 Patient 2

Patient 2 is a 4-year and 6 months-old-girl. She was found to have developmental delay for 3 years and had facial weakness for 1 year. She cannot frown, cannot chew her cheeks and was expressionless when laughing and crying. Her bilateral forehead lines and nasolabial fold disappeared. There was no related family history.

**Figure 3:**
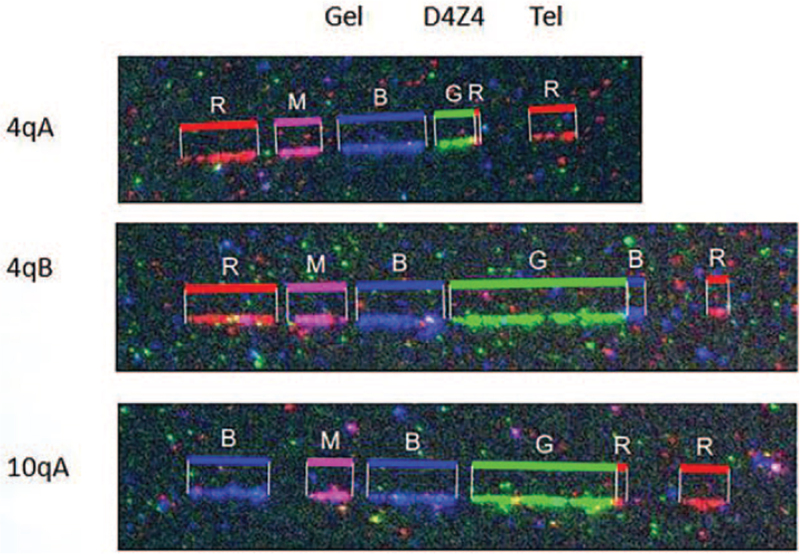
The patient2's molecular combing pattern of a D4Z4 repeat contraction on 4qA. The color code for the contracted 4qA allele from centromere to telomere corresponds to red, magenta, blue, green, red, and red. The first line showed a contracted size 4qA allele with 3 D4Z4 (green region) repeats (9 kb).The second and third rows show that the 4qB and 10qA alleles are of normal size.

There were no deformities of the spine and limbs. Muscle strength of the limbs was decreased slightly, muscle tension was decreased, bilateral knee tendon reflexes could be elicited, and pathological signs were not elicited. Attention deficit hyperactivity disorder is possible, and her head MRI shows arachnoid cysts. Her electroencephalogram was normal. The newborn hearing screening of patient 2 failed, and she was wearing a hearing aid by the age of 1 year and 1 month. Her electrocardiogram (ECG) showed sinus tachycardia. Echocardiography showed oval foramen ovale and tricuspid regurgitation. Molecular combing of the FSHD1 locus showed a contracted size 4qA allele with 3 D4Z4 repeats (9 kb), which led to a diagnosis of FSHD1 (shown in Fig. 3). Molecular tests of her parents were normal. The patient received cocktail therapy (vitamin B1 tablets, vitamin B2 tablets, vitamin B6 tablets, vitamin C tablets, vitamin E tablets, idebenone tablets, etc.) However, her symptoms did not improve during follow-up one month after discharge. According to her recent follow-up, the patient's main symptoms are facial weakness and proximal muscle weakness.

## 3 Discussion and conclusion

Facioscapulohumeral muscular dystrophy (FSHD) is a slowly progressive muscular dystrophy. Currently, there are 2 genetically distinct types of FSHD, FSHD1, and FSHD2.^[5]^ Facioscapulohumeral muscular dystrophy type 1 (FSHD1, OMIM: 158900) is an autosomal dominant genetic disease in which integral copies of the 3.3 kb tandem repeat unit D4Z4 are deleted in the subtelomere region of chromosome 4q35.41. In FSHD1 patients, there are only 1 to 10 copies of D4Z4 repeats at the 4q35 region, while 11 to 100 copies are found in the normal population.^[6–8]^ Some studies have shown that there is a negative correlation between the number of D4Z4 repetitions and the severity of FSHD. Therefore, studies have shown that very short alleles (1–3 D4Z4 repeats) are related to the most severe form of the disease.^[7,8]^ The D4Z4 repeat sequence contains the DUX4 gene, which is abnormally expressed in skeletal muscle cells of FSHD patients. The abnormal expression of DUX4 leads to dysregulation of molecular pathways that are involved in muscle differentiation, oxidative stress responses, immune responses, and protein turnover. However, the precise mechanism leading to FSHD is still unknown.^[3,9,10]^

Facioscapulohumeral muscular dystrophy type 2 (FSHD2, OMIM: 158901) is associated with mutations of the structural maintenance of the chromosome flexible hinge domain-containing protein 1^[11]^ (SMCHD1) gene on chromosome 18p1143 or the DNA methyltransferase 3B^[12]^ (DNMT3B) gene on chromosome 20q11 in the presence of a disease-permissive 4qA haplotype. Both types of mutations are associated with insufficient epigenetic repression of D4Z4 repeats, leading to aberrant expression of double homeobox protein 4 (DUX4) in skeletal muscles and consequently, disease progression. FSHD2 can present with autosomal dominant or recessive inheritance.^[3,5]^

We used facioscapulohumeral muscular dystrophy as a key word to search the PubMed database for cases of infantile or early-onset FSHD that have previously been reported and summarized the reported clinical features of early-onset FSHD. To date, we identified a total of 22 articles^[2,5,13–32]^ with individual data on 324 patients. We have summarized the characteristics of the 324 cases reported in the literature in Table 1. Among the 324 patients, age at assessment ranged from 7 months to 72 years. We found that 74/324 patients (22.8%) showed symptoms in the first year of life, mainly facial weakness resulting in feeding difficulties and less frequently inadequate eye closure. In general, the pattern of early-onset FSHD muscle involvement is similar to the typical FSHD phenotype. Systemic features were reported in most of the patients and included spinal deformities (42.0%), hearing loss (39.1%), vision loss (5.6%), retinopathy (18.2%), epilepsy (7.4%), developmental delay (5.9%), mental retardation (10.5%), and pulmonary and cardiac abnormalities. Almost half (114/324, 35.1%) of the patients were wheelchair dependent. In general, early-onset FSHD can be described as a severe subtype associated with severe muscle weakness and frequent systemic features, including epilepsy, hearing difficulties, retinal abnormalities (Coats syndrome), intellectual disability, and pulmonary and cardiac abnormalities.

**Table 1:** Previously reported patient characteristics of early-onset FSHD cases (n = 324).

Characteristics	
Age at reporting	7 m–72 y
Male sex (number/total number)	147/324 (45.4%)
Age at onset in the first year of life(number/total number)	74/324 (22.8%)
Diagnosed before 5 yr old (number/total number)	24/324 (7.4%)
Diagnosed before 10 years old (number/total number)	44/324 (13.6%)
First symptom (number)	facial weakness (226), hearing loss 7)
FSHD1/FSHD2 (number)	323/1
Familial/Sporadic	89/128
Wheelchair dependency (number/total number)	114/324 (35.1%)
Hearing loss (number/total number)	127/324 (39.1%)
Vision loss	18/324 (5.6%)
Retinopathy	59/324 (18.2%)
Decreased forced vital capacity (%FVC)	48/324 (14.8%)
Epilepsy	24/324 (7.4%)
Spinal deformities	136/324 (42.0%)
Development delay	19/324 (5.9%)
Mental retardation	34/324 (10.5%)
ECG abnormalities	9/324 (2.8%)
Non-invasive ventilation	26/324 (8.0%)
Diagnose	Southern blot analysis (244), DNA methylation studies (1)
1–3 D4Z4 repeat	73

ECG = electrocardiograph, FSHD1 = facioscapulohumeral muscular dystrophy type 1, FSHD2 = facioscapulohumeral muscular dystrophy type 2, FVC = forced vital capacity.

In the classical FSHD phenotype, FSHD can affect almost any skeletal muscle but typically spares extraocular muscles, cardiac muscles, and bulbar muscles. Extramuscular manifestations are rarely symptomatic. Symptomatic retinal vasculopathy (Coats syndrome) is seen in approximately 1% of FSHD patients, and patients with large D4Z4 deletions are more typical.^[33]^ However, we found that 18.2% of early-onset FSHD had retinopathy. The differences between patients with early-onset and adult-onset FSHD were more severe muscle weakness, more rapid progression and more frequently occurring systemic features. In addition, some systemic features are associated with only early-onset FSHD, such as mental retardation and epilepsy.^[5]^ If a patient is in infancy with bifacial weakness, a weak suck reflex, gagging, an expressionless face, and, later on, an inability to close the eyes, smile, whistle, or speak clearly, they may have FSHD. There are also systemic manifestations such as congenital sensorineural hearing loss and retinal diseases, and especially when family members are diagnosed with FSHD, we need to suspect that these may be signs of early-onset FSHD.

Clinical criteria for the diagnosis of FSHD include the presence of characteristic findings and the absence of other explanations. FSHD is suggested by the presence of facial weakness and weakness of the shoulder scapular stabilizers or foot dorsiflexors. There is an absence of: ptosis, weakness of extraocular muscles or bulbar weakness. Electromyography in a patient or affected family member will show myotonia or neurogenic changes.^[33]^ In general, there is no correlation between muscle biopsy or electromyographic findings and the severity of muscle involvement. Apart from confirmation of the myopathic process, these investigations do not contribute to the diagnosis and do not allow for prediction of the disease course. Because muscle biopsy lacks specificity, it is mostly a clinical diagnosis.^[34]^

At present, genetic methods can be used for diagnosis and typing. Based on typical clinical features and an inheritance pattern consistent with autosomal dominant inheritance, together with an affected first-degree relative with a genetically confirmed diagnosis, genetic testing is not necessary for each affected individual.^[5,33]^ The standard diagnostic method for FSHD1 is genetic, including the use of Southern blots or molecular combing methods to determine the size of the D4Z4 repeat on chromosome 4q. The diagnostic method for FSHD2 is the identification of hypomethylation of the D4Z4 repeat array in the subtelomeric region of chromosome 4q35 on a chromosome 4 permissive haplotype. The FSHD2 genetic diagnosis is even more complicated because it requires the identification of a permissive allele together with a pathogenic mutation in the SMCHD1 gene. The standard genetic test to identify mutations in SMCHD1 is based on next-generation sequencing (NGS).^[3–5,34,35]^ Currently, commercial genetic testing for FSHD is limited to FSHD1.^[35]^

Molecular genetic testing approaches can include targeted analysis for the repeat size of the D4Z4 repeat array in the subtelomeric region of chromosome 4q35 and haplotype analysis, DNA methylation studies, and single-gene testing.^[34–37]^ Targeted testing is typically performed by Southern blotting, but molecular combing techniques have also been described. Molecular combing has a higher resolution than Southern blotting. Molecular combing has superior analytical validity compared to Southern blot for determining D4Z4 contraction size, detecting mosaicism, and resolving borderline and indeterminate Southern blot results.^[36]^ Haplotype analysis is recommended concurrently with testing for a D4Z4 contraction to determine if an abnormal allele is present on a permissive or nonpermissive haplotype distal to the last D4Z4 repeat.^[34–37]^ In individuals who do not have a contracted D4Z4 repeat array identified and have at least 1 repeat array with a permissive chromosome 4 haplotype, D4Z4 methylation analysis should be done next. D4Z4 hypomethylation suggests the presence of a heterozygous SMCHD1 or DNMT3B pathogenic variant. Sequence analysis of SMCHD1 and DNMT3B should be done in individuals with D4Z4 hypomethylation to detect small intragenic deletions/insertions and missense, nonsense, and splice site variants.^[34–37]^ At present, the prenatal diagnosis of FSHD uses Southern blotting, but this method requires a large amount of high-quality DNA, the culture of amniotic fluid or villi cells, and the experimental procedures are complicated.^[34–37]^ We have explored the new method of Bionano optical mapping combined with karyomapping as a new strategy for prenatal diagnosis. Karyomap gene chips use high-density SNP loci to determine the genome-wide parental haplotype, which provides a more comprehensive method for linkage analysis of single-gene diseases.^[34–37]^

There is presently no effective pharmacological treatment for FSHD. In recent years, with the understanding of the molecular mechanism of FSHD, we have focused on epigenetic and transcriptional regulation of the aberrantly expressed DUX4 in FSHD. There are 2 future treatment strategies for FSHD:

1. therapies to increase muscle bulk or strength (anabolic agents, myostatin or follistatin inhibitors) and at present, ACE-083 TGF-β protein-related targeted therapy for activin and myostatin (GDF8) are undergoing clinical trials.
2. Therapies to halt disease progression (molecular knockdown of DUX4 or downstream targets of DUX4): some gene-silencing strategies are being evaluated in clinical trials (such as eteplirsen and nusinersen).

Facio therapies announced that more than 300 compounds could inhibit the production of myotoxic DUX4 protein.^[4,33]^ Because of the systemic symptoms of FSHD, we should treat the clinical features symptomatically. There are a range of treatments for extramuscular manifestations, such as rehabilitation training, speech therapy, installation of ankle or foot orthoses, surgical treatment of spinal deformities, management of chronic pain by physical therapy and medication, monitoring respiratory function, lubricants to prevent drying of the sclera or taping the eyes shut during sleep to treat exposure keratitis, treatment for retinal vasculopathy as per an ophthalmologist, and standard treatment of sensorineural hearing loss.^[35]^

We report 2 pediatric patients with FSHD type 1. Summarizing the characteristics of our 2 pediatric cases of FSHD, their main symptoms are facial weakness and proximal muscle weakness, patient1 is family-associated and patient 2 is sporadic. The biopsy shows no specific changes. The pattern of muscle involvement in patients are similar to the classical FSHD phenotype, their age of onset matched early-onset FSHD, and both of them have systemic features(e.g., spinal deformities, development delay, hearing loss or ECG abnormalities). According to clinical manifestations and accurate genetic testing, we can get a definite diagnosis.

In conclusion, early-onset FSHD has severe muscle weakness and frequent systemic features, which can be described as a severe subtype of FSHD. We suggest that if a 0 to 5-year-old child shows poor facial expression or other facial weakness (facial asymmetry, facial diplegia, infantile sucking difficulties, indistinct speech, drooling), especially if they have a family history (including myopathy, hearing loss and retinopathy), FSHD should be included in the differential diagnosis, and therefore Southern blot analysis or molecular combing should be performed. Although FSHD has made great progress in pathogenesis and diagnostic methods, there are still no effective pharmacological treatments and many problems need to be solved. The gene therapy of FSHD is being studied. For these patients, early identification and genetic diagnosis should be carried out to improve the prognosis of patients with precision therapy.

## Author contributions

**Data curation:** Haiyan Yang, Siyi Gan.

**Writing – original draft:** Ting Xiao.

**Writing – review & editing:** Liwen Wu.
